# Supplementary material for: HIVprotI: an integrated web based platform for prediction and design of HIV proteins inhibitors
Source: J Cheminform. 2018 Mar 9;10:12. doi: 10.1186/s13321-018-0266-y (PMC5845081; doi:10.1186/s13321-018-0266-y)
Supplement: Supplementary file 2 — Additional file 2. Source code of HIVProtI web server. [file 13321_2018_266_MOESM2_ESM.zip › HIVprotI_Source-code/hivprotI/weka-3-6-6/doc/weka/classifiers/trees/j48/Stats.html]

Stats


---


|  |  |  |
| --- | --- | --- |
| **Overview** \| **Package** \| **Class** \| **Tree** \| **Deprecated** \| **Index** \| **Help** \| **Weka's home** | |  |
| **PREV CLASS**   NEXT CLASS | **FRAMES**    **NO FRAMES**     **All Classes** |  |
| SUMMARY: NESTED \| FIELD \| CONSTR \| METHOD | DETAIL: FIELD \| CONSTR \| METHOD |  |


---


## weka.classifiers.trees.j48 Class Stats

```
java.lang.Object
  weka.classifiers.trees.j48.Stats
```

**All Implemented Interfaces:**: RevisionHandler

---

``` public class Stats extends java.lang.Object implements RevisionHandler ```

Class implementing a statistical routine needed by J48 to
compute its error estimate.

**Version:**
:   $Revision: 1.9 $

**Author:**
:   Eibe Frank (eibe@cs.waikato.ac.nz)

---

**Constructor Summary**

`Stats()`


| **Method Summary** | |
| --- | --- |
| `static double` | `addErrs(double N, double e, float CF)`             Computes estimated extra error for given total number of instances and error using normal approximation to binomial distribution (and continuity correction). |
| `java.lang.String` | `getRevision()`             Returns the revision string. |

**Methods inherited from class java.lang.Object**

`equals, getClass, hashCode, notify, notifyAll, toString, wait, wait, wait`

**Constructor Detail**

### Stats

```
public Stats()
```


**Method Detail**

### addErrs

```
public static double addErrs(double N,
                             double e,
                             float CF)
```

:   Computes estimated extra error for given total number of instances
    and error using normal approximation to binomial distribution
    (and continuity correction).

    :   **Parameters:**: `N` - number of instances: `e` - observed error: `CF` - confidence value

---


### getRevision

```
public java.lang.String getRevision()
```

:   Returns the revision string.

    :   **Specified by:**: `getRevision` in interface `RevisionHandler`
    :   **Returns:**: the revision


---


|  |  |  |
| --- | --- | --- |
| **Overview** \| **Package** \| **Class** \| **Tree** \| **Deprecated** \| **Index** \| **Help** \| **Weka's home** | |  |
| **PREV CLASS**   NEXT CLASS | **FRAMES**    **NO FRAMES**     **All Classes** |  |
| SUMMARY: NESTED \| FIELD \| CONSTR \| METHOD | DETAIL: FIELD \| CONSTR \| METHOD |  |


---
